# Phytochemical Study of *Tapirira guianensis* Leaves Guided by Vasodilatory and Antioxidant Activities

**DOI:** 10.3390/molecules22020304

**Published:** 2017-02-18

**Authors:** Amélia M. G. Rodrigues, Denise O. Guimarães, Tatiana U. P. Konno, Luzineide W. Tinoco, Thiago Barth, Fernando A. Aguiar, Norberto P. Lopes, Ivana C. R. Leal, Juliana M. Raimundo, Michelle F. Muzitano

**Affiliations:** 1 Laboratório de Biologia do Reconhecer, Centro de Biociências e Biotecnologia, Universidade Estadual do Norte Fluminense Darcy Ribeiro, Av. Alberto Lamego, 2000, Parque Califórnia, Campos dos Goytacazes, 28013-602 Rio de Janeiro, Brazil; amelia.fmc@gmail.com; 2 Laboratório Integrado de Pesquisa, Universidade Federal do Rio de Janeiro, Campus Macaé, Av. Aluízio da Silva Gomes, 50, Novo Cavaleiros, Macaé, 27930-560 Rio de Janeiro, Brazil; 3 Laboratório de Produtos Bioativos, Universidade Federal do Rio de Janeiro, Campus Macaé, Polo Novo Cavaleiro—IMCT, R. Alcides da Conceição, 159, Novo Cavaleiros, Macaé, 27933-378 Rio de Janeiro, Brazil; deololiveira@gmail.com (D.O.G.); barththiago@yahoo.com.br (T.B.); 4 Núcleo de Estudos em Ecologia e Desenvolvimento Sócio-Ambiental de Macaé, Universidade Federal do Rio de Janeiro, Av. São José Barreto, 764—São José do Barreto. Macaé, 27965-045 Rio de Janeiro, Brazil; tkonno@uol.com.br; 5 Instituto de Pesquisa de Produtos Naturais Walter Mors, Centro de Ciências da Saúde, Universidade Federal do Rio de Janeiro, Brazil; luzitinoco@hotmail.com; 6 Núcleo de Pesquisa em Produtos Naturais e Sintéticos, Departamento de Física e Química, Faculdade de Ciências Farmacêuticas de Ribeirão Preto, Universidade de São Paulo, Av. do Café s/n. 14040-020 Ribeirão Preto, Brazil; fndarmani@gmail.com (F.A.A.); npelopes@fcfrp.usp.br (N.P.L.); 7 Laboratório de Química, Universidade Federal do Rio de Janeiro—Campus Macaé, Av. Aluízio da Silva Gomes, 50, Novo Cavaleiros. Macaé, 27930-560 Rio de Janeiro, Brazil; 8 Laboratório de Produtos Naturais e Ensaios Biológicos, Departamento De Produtos Naturais e Alimentos, Faculdade de Farmácia, Universidade Federal do Rio de Janeiro, 21941-902 Rio de Janeiro, Brazil; ivanafarma@yahoo.com.br

**Keywords:** *Tapirira guianensis*, aorta, vasodilation, antioxidant, tannin, flavonoid

## Abstract

The aim of this research was to perform a phytochemical study of the methanol leaves extract of *T. guianensis* (MET) guided by vasodilatory and antioxidant activities. The chemical profile of MET and the ethyl acetate fraction (EA fraction) was determined by HPLC-UV-MS and EA fraction guided fractionation by reverse-phase chromatography. The vasorelaxant effects of MET, fractions, sub-fractions and constituents were assessed on rat aorta pre-contracted with phenylephrine. Antioxidant activity was evaluated by using a DPPH assay. The results show that MET-induced vasodilation was dependent on NO/cGMP; and that the PI3K/Akt pathway seems to be the main route involved in eNOS activation. The EA fraction showed greater vasodilatory and antioxidant potency and was submitted to further fractionation. This allowed the isolation and characterization of quercetin, quercetin 3-*O*-(6″-*O*-galloyl)-β-d-galactopyranoside and 1,4,6-tri-*O*-galloyl-β-d-glucose. Also, galloyl-HHDP-hexoside and myricetin deoxyhexoside were identified by HPLC-UV-MS. These compounds are being described for the first time for *T. guianensis*. 1,4,6-tri-*O*-galloyl-β-d-glucose and quercetin 3-*O*-(6″-*O*-galloyl)-β-d-galactopyranoside showed no vasodilatory activity. Quercetin and myricetin glycoside seems to contribute to the MET activity, since they have been reported as vasodilatory flavonoids. MET-induced vasodilation could contribute to the hypotensive effect of *T. guianensis* previously reported.

## 1. Introduction

Hypertension is one of the key risk factors for cardiovascular diseases, which are the main cause of death worldwide [1]. Hypertension is characterized by a chronic elevation of arterial blood pressure, in which abnormally increased vascular tone plays a major role in the maintenance of high blood pressure [1].

The endothelium regulates the contractility of vascular smooth muscle by releasing relaxing and contracting factors and the loss of its normal function results in increased vascular tone [2]. Endothelial dysfunction is a common feature of hypertensive patients, a condition that includes reduced endothelium-dependent vasodilation, morphological changes of vascular smooth muscle and a hypercoagulatory state [3,4,5]. Elevated levels of reactive oxygen species are a key player in the pathogenesis of endothelium dysfunction leading to decreased nitric oxide (NO) bioavailability, the main endothelium relaxant factor, and endothelial NO synthase (eNOS) uncoupling [5,6].

Phenolic compounds are plant secondary metabolites widely distributed in Nature that are known for their beneficial effects in many processes involved in the pathogenesis of cardiovascular diseases. Epidemiological assessments show reduction in the incidence of these diseases, besides beneficial effects observed in preclinical and clinical studies. Phenolic compounds have been related to vasodilatory activity [7], hypotensive effect [8,9], improvement of endothelial dysfunction, reduction of oxidative stress [10,11,12] and attenuation of vascular aging [13]. Moreover, the cardiovascular action of herbal and non-herbal products is largely attributed to the presence of phenolic compounds [12].

*Tapirira* genus (Anacardiaceae) is composed of approximately 40 species mainly distributed in South America [14]. Among them, *Tapirira guianensis* Aubl. species is popularly known in Brazil as “pau-pombo” or “tapiririca”. It is used in traditional medicine against leprosy, diarrhea and syphilis [15]. Moreover, in Brazilian Amazonian coastal areas, the natural wood and an inner bark mixture of *T. guianensis* are used for the treatment of infant oral thrush and throat and mouth sore, respectively [16].

Phytochemical studies have reported the isolation of different secondary metabolites from *T. guianensis*, some with already described biological activities. David et al. [15] reported the presence of β-sitosterol, in addition with two new compounds identified as 2-[10(*Z*)-heptadecenyl]-1,4-hydroquinone and (4*R*,6*R*)-dihydroxy-4-[10(*Z*)-heptadecenyl]-2-cyclohexenone in a methanol extract of the seeds, which showed cytotoxic activity against different human cancer cell lines. Flavonoids, norisoprenoids and terpenes have been isolated from *T. guianensis* leaves, including the flavonoids kaempferol 3-α-rhamnoside, kaempferol-3-α-arabinofuranoside, quercetin-3-α-rhamnoside and kaempferol [17]. Roumy et al. [18] identified new cyclic alkyl polyol derivatives from *T. guianensis* bark with anti-protozoal and anti-bacterial activities.

The ethanol extract of leaves and twigs of *T. guianensis* induced a 38.5% reduction of arterial blood pressure in anaesthetized rats, indicating its potential as a hypotensive agent. This effect could be related to the presence of triterpenoids and flavonoids in the extract [19]. However, there are no studies about the mechanisms involved in the cardiovascular action of *T. guianensis*, as well as about the compounds responsible for that effect. Thus, the aim of the present study was to investigate the vasodilatory and antioxidant activities of the methanol extract of *T. guianensis* (MET) and to identify the compounds involved in these effects.

## 2. Results

### 2.1. Chemical Analysis of MET

**Figure 1:**
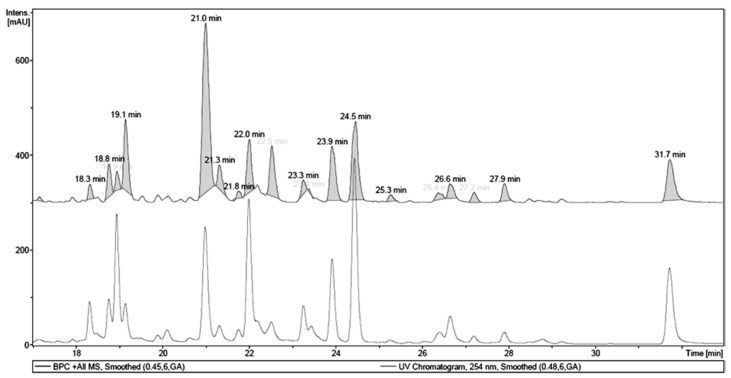
LC-UV-MS chromatogram of the ethyl acetate fraction of the methanol extract of *T. guianensis* leaves.

**Figure 2:**
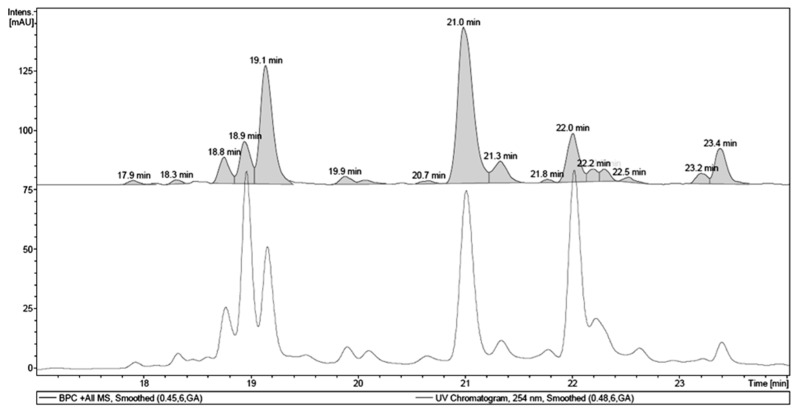
LC-UV-MS chromatogram of sub-fraction 4 from the ethyl acetate fraction of the methanol extract of *T. guianensis* leaves.

Initially, MET was fractionated affording a main active fraction obtained by partition with EA. The EA fraction was analyzed by LC-UV-MS (Figure 1) and showed a very similar phenolic profile when compared to MET. Figure 2 shows UV and MS chromatograms of EA, where the main UV chromatogram peaks, *t_R_* 21.0, 22.0, 23.9, 24.5 and 31.7 min, showed a characteristic flavonol UV spectrum, with λ_MAX_ at 250–280 and 350–380 nm [20], with the exception of the peak at 18.9 min. MS analyses of each main peak allowed the identification of hydrolysable tannins and flavonols, specifically quercetin and myricetin glycosides, as shown in Figure 1 and Table 1.

Chromatographic fractionation of the EA fraction afforded four phenolic sub-fractions: 3, 4, 5 and 6. Two of them, 3 and 6, corresponded to pure compounds, the hydrolysable tannin 1,4,6-tri-*O*-galloyl-β-d-glucopyranose (EA1) and the flavonoid quercetin (EA3), respectively. From sub-fraction 4, the flavonoid 3-*O*-(6″-*O*-galloyl)-β-d-galactopyranoside (EA2) was isolated and characterized In addition, sub-fraction 4 was also analyzed by LC-UV-MS. Thus, it was possible to identify the isolated compounds EA1 and EA2 and other major components: one hydrolysable tannin, galloyl-HHDP-hexoside, and one flavonoid, myricetin deoxyhexoside (Figure 2 and Table 2).

### 2.2. Vasodilatory Effect of MET

**Figure 3:**
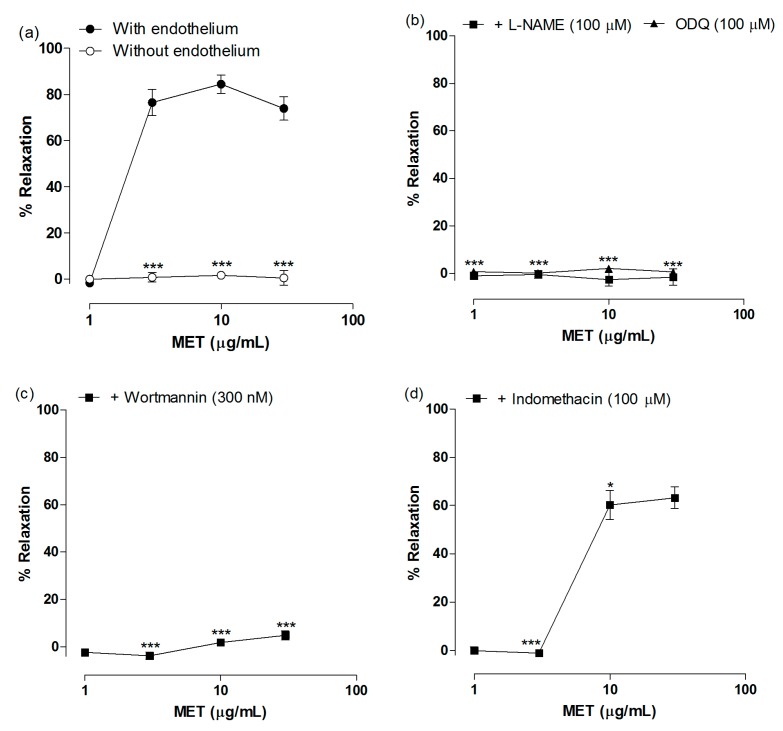
Mechanism of action of vasodilatory activity of the methanol extract of *T. guianensis* leaves (MET). (**a**) Concentration-response curves for MET in aortas with and without endothelium; (**b**) Effect of MET in endothelium-intact rings pretreated with L-NAME (100 μM) or ODQ (100 μM); (**c**) Effect of MET in endothelium-intact rings pretreated with wortmannin (300 nM); (**d**) Effect of MET in endothelium-intact rings pretreated with indomethacin (10 μM). Data are mean ± standard error of mean (S.E.M.) (*n* = 5–7). * *p* < 0.05 and *** *p* < 0.0001 compared to with endothelium.

MET induced intense relaxation of aortic rings with endothelium pre-contracted with Phe. At 10 µg/mL, MET produced a relaxation of 84.46% ± 4.0% (Figure 3a). The concentration of MET necessary to reduce by 50% Phe-induced contraction of aorta (IC_50_) was 4.46 ± 0.97 µg/mL. To test whether the vasorelaxation induced by MET was endothelium-dependent, it was tested in endothelium-denuded aortic rings.

Removal of endothelium completely inhibited MET-induced vasodilation (Figure 3a). Thus, we evaluated the involvement of the endothelium-derived factors NO and PGI_2_. Pretreatment of aortic rings with L-NAME and ODQ abolished MET-induced vasodilation, indicating that the NO/cGMP pathway is crucial for the effect (Figure 3b). Moreover, similar results were obtained with the pretreatment with wortmannin (Figure 3c), indicating that MET induces eNOS activation mainly via activation of PI3K/Akt pathway and eNOS phosphorylation. Indomethacin induced a rightward shift of MET concentration-response curve (Figure 3d), demonstrating that the extract also acts through the activation of PGI_2_ production.

### 2.3. Vasodilatory Effect of Fractions, Sub-Fractions and Isolated Compounds

**Figure 4:**
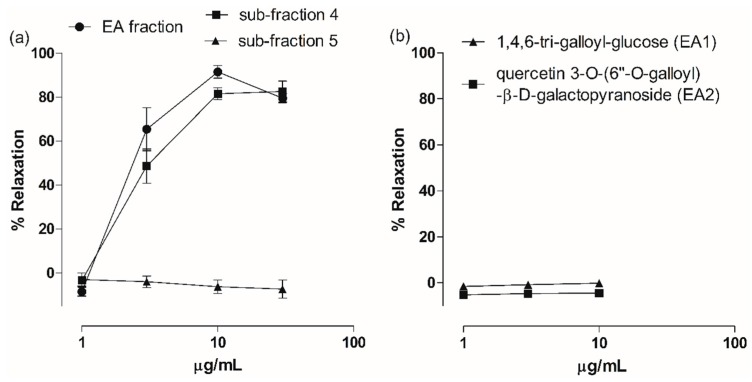
Effects of ethyl acetate fraction (EA fraction), sub-fractions and isolated compounds on aortic rings with endothelium. (**a**) Concentration-response curves for EA fraction, sub-fraction 4 and sub-fraction 5; (**b**) Concentration-response curves for 1,4,6-tri-*O*-galloyl-glucose (EA1) and quercetin 3-*O*-(6″-*O*-galloyl)-β-d-galactopyranoside (EA2). Data are mean ± S.E.M. (*n* = 5–6).

All fractions obtained from liquid-liquid partitioning of MET were evaluated in aortas with endothelium and EA fraction was the most effective in producing vasodilation. At 30 µg/mL, the DCM and EA fractions induced 53.56% ± 5.87% and 79.52% ± 2.86% relaxation (Figure 4a). The EA fraction (IC_50_ 3.09 ± 0.37 µg/mL) exhibited higher potency than the DCM fraction (IC_50_ 44.81 ± 6.77 µg/mL). Vascular relaxation induced by HN, BT and Aq fractions was inferior to 50%.

Sub-fraction 4 was evaluated and produced significant relaxation of rat aorta, similar to EA fraction-induced vasodilation. At 10 µg/mL, sub-fraction 4 induced 81.53% ± 2.70% of relaxation (IC_50_ 3.99 ± 0.70 µg/mL) (Figure 4a). On the other hand, sub-fraction 5 had no vasodilator effect. In addition, the isolated hydrolysable tannin EA1 and the flavonoid EA2 were evaluated because their vasodilatory activity is not described in literature yet. As shown in Figure 4b, they had no effect on aorta contractility.

### 2.4. Antioxidant Effect of MET and Fractions

In vitro antioxidant activity of MET and its fractions were evaluated by using the DPPH assay. *Ginkgo biloba* extract Egb 761^®^, with an EC_50_ of 22.91 ± 0.66 µg/mL (Table 3) was used as positive control. MET and its fractions showed antioxidant activity with different potency (Table 3). MET (EC_50_ 3.12 ± 0.20 µg/mL) exhibited the highest potency, while EA fraction (EC_50_ 5.33 ± 0.16 µg/mL) and BT fraction (EC_50_ 6.05 ± 0.19 µg/mL) were the most potent fractions. MET and all fractions exhibited greater potency than Egb 761^®^.

## 3. Discussion

Here we have presented a phytochemical study guided by vasodilatory and antioxidant activities of a methanol leaves extract of *T. guianensis*. Phytochemical analyses revealed that MET is a phenolic-rich extract, containing tannins and flavonoids. The compounds isolated and characterized by ^1^H and ^13^C-NMR were 1,4,6-tri-*O*-galloyl-β-d-glucose (EA1), quercetin 3-*O*-(6″-*O*-galloy)-β-d-galactopyranoside (EA2) and quercetin (EA3). Also, galloyl-HHDP-hexoside and myricetin deoxyhexoside were identified by LC-UV-MS. As far as we know, this is the first report of the occurrence of these compounds in *T. guianensis*.

Although no ethnopharmacological studies describing the medicinal use of *T. guianensis* for cardiovascular diseases have been found, one animal study showed promising results [19]. The ethanol extract of leaves from *T. guianensis* was able to reduce blood pressure in Sprague-Dawley rats, probably due the action of triterpenoids and flavonoids presents in the extract [19]. Our data showed that MET induces intense vasodilation in Wistar rat aortic rings, which could be, at least in part, responsible for the hypotensive effect described by Jiménez et al. [19].

MET-induced vasodilation was endothelium-dependent, since it was abolished by removal of the endothelium. Vascular endothelial cells produce and release relaxant factors, NO, PGI_2_ and endothelium-derived hyperpolarizing factor (EDHF), which play an important role in the regulation of vascular smooth muscle tone [21,22]. The contribution of NO, PGI_2_ and EDHF for vascular relaxation is heterogeneous and varies according to the size of the blood vessel [22,23]. NO has a more pronounced role in large arteries, such as aorta and epicardial coronary arteries, while the contribution of PGI_2_ does not change according to the type of vessel and EDHF is more important in the control of vessel diameter in smaller arteries and arterioles [22,23].

Considering endothelium-dependent mechanisms, most of vasodilator compounds isolated from plants produce vasodilation by activating the NO/cGMP pathway, whereas PGI_2_ plays a minor role in the mechanism of action of these compounds [7]. MET-induced vasodilation was completely inhibited in the presence of L-NAME and OQD, while indomethacin partially inhibited MET effect, indicating that vascular relaxation was mediated predominantly by NO.

Classically, eNOS activation is dependent on intracellular calcium concentration increase, as occurs when acetylcholine and histamine activates endothelial receptors. However, eNOS can also be activated by phosphorylation on specific amino acids residues. Some agonists such as estrogen and insulin induce vasodilation by activating the PI3K/Akt pathway, with subsequent phosphorylation of eNOS on Ser1177 [24]. MET seems to induce vasodilation by activating the PI3K/Akt pathway, since its effect was significantly blocked in aortic rings pretreated with wortmannin.

Among MET fractions, EA fraction presented greater vasodilatory and antioxidant potency and was submitted to further fractioning. Sub-fraction 4 showed a vasodilator profile similar to EA fraction, while sub-fractions 3 (EA1) and 5 were not active. Sub-fraction 6 was identified as the pure compound quercetin (EA3), extensively studied for its pharmacological properties. Quercetin was shown to produce both endothelium-dependent, with the involvement of NO and PGI_2_, and endothelium-independent vasodilation [25,26,27]. Thus, this flavonol is, at least partially, responsible for the vasodilatory activity of MET.

Fractionation of sub-fraction 4 resulted in the isolation of quercetin 3-*O*-(6″-*O*-galloyl)-β-d-galactopyranoside (EA2). LC-MS analysis showed that the isolated hydrolysable tannin 1,4,6-tri-*O*-galloyl-β-d-glucose (EA1, sub-fraction 3) is also present in sub-fraction 4. However, both of them did not present vasodilatory activity in rat aorta. Galloyl tannins and flavonoid galloyl glycosides have been shown to induce vasodilation and to inhibit the activity of angiotensin converting enzyme [28,29,30]. Penta-*O*-galloyl-β-glucoside induced NO-mediated vascular relaxation of rat aorta [8] and quercetin 3-*O*-β-d-galactopyranoside (hyperoside) showed vasodilatory activity in rat basilar artery [30], suggesting that the vasodilation effect is influenced by the number and the position of galloyl groups both in tannins and flavonoids [31,32].

In addition, galloyl-HHDP-hexoside and myricetin deoxyhexoside were identified in sub-fraction 4. Myricetin deoxyhexoside is a glycoside of myricetin, usually found in plants as myricetin-3-*O*-rhamnoside, also known as myricitrin. It was shown to attenuate endothelial cells apoptosis through PI3K/Akt signaling [33,34], but no vasodilatory activity has already been described. Also, part of the antihypertensive action of *Tetraclinis articulates* (Cupressaceae) was assigned to myricitrin [35].

Besides vascular effects, phenolic compounds are well known antioxidant agents, which is of particular interest since oxidative stress is a common feature of cardiovascular diseases [36]. MET and EAF showed a potent in vitro ability to scavenge the free radical DPPH, that could be attributed to the phenolic compounds identified. 1,4,6-tri-*O*-galloyl-β-d-glucose (EA1), quercetin 3-*O*-(6″-*O*-galloy)-β-d-galactopyranoside (EA2), quercetin (EA3) and myricitrin have already been shown to have antioxidant activity [37,38,39,40]. In addition, antioxidant effect of quercetin and its metabolites was described in vascular smooth muscle cells from normotensive and spontaneously hypertensive rats, by inhibiting the membrane NADPH oxidase activity [41].

## 4. Materials and Methods

### 4.1. Plant Material and Preparation of Crude Extract and Fractions

Leaves of *Tapirira guianensis* (Anacardiaceae) were collected on January 2012 at Parque Nacional da Restinga de Jurubatiba, Quissamã, Rio de Janeiro, Brazil, under legal authorization (SISBIO 39673-2). Botanical identification was performed by Dr. Tatiana Ungaretti Paleo Konno and a voucher specimen was deposited at the Universidade Federal do Rio de Janeiro Herbarium under the number RFA38757. *T. guianensis* leaves (3 Kg) were dried, triturated and extracted with methanol by maceration to yield the total dry crude extract MET (320.20 g), i.e., dry extract yield of 10.67% (*w*/*w*). A sample (50 g) of MET was solubilized in MeOH/distilled water (9:1) in agitation. The solution obtained was submitted to successive partitions with organic solvents in the following order: *n*-hexane (HN; eight partitions; 6.5287 g), dichloromethane (DCM; four partitions; 1.7312 g), ethyl acetate (EA; 11 partitions; 26.1552 g) and *n*-butanol (BT; four partitions; 7.2052 g). After partition with *n*-butanol the remaining aqueous fraction (Aqr; 6.0103 g) was obtained and it was submitted to lyophilization.

### 4.2. Chromatographic Separation of EA Fraction 

**Figure 5:**
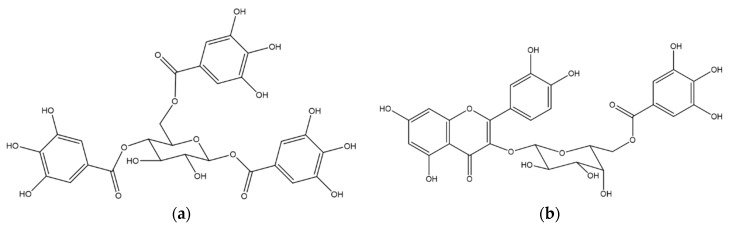
Chemical structure of compounds isolated from *T. guianensis* leaves. (**a**) 1,4,6-tri-*O*-galloyl-β-d-glucose (EA1); (**b**) quercetin 3-*O*-(6″-*O*-galloyl)-β-d-galactopyranoside (EA2).

An aliquot of EA fraction (2 g) was re-suspended in distilled water and chromatographed (H_2_O/MeOH gradient) on silanized silica column (0.063–0.200 mm, Merck, Darmstadt, Germany) affording eight sub-fractions, four of them, 3, 4, 5 and 6, presented UV chromatographic profiles characteristic of phenolic compounds. Sub-fraction 3, containing one pure compound codified as EA1 (10.1 mg), eluted with H_2_O/MeOH 20%, and was identified as the hydrolysable tannin 1,4,6-tri-*O*-galloyl-β-d-glucose (Figure 5a) by ^1^H- and ^13^C-NMR in comparison with the literature [42]. The semi-pure fraction sub-fraction 4 (100.7 mg) was re-suspended in distilled water and was purified on an RP-18 silanized silica (40–63 µM, Merck^®^; H_2_O/MeOH gradient) affording one isolated flavonoid, codified as EA2 (9.6 mg), eluted with H_2_O/MeOH 40%, and identified as quercetin 3-*O*-(6″-*O*-galloy)-β-d-galactopyranoside (Figure 5b) by ^1^H- and ^13^C-NMR in comparison with the literature [43]. Sub-fraction 6, containing one pure compound, codified as EA3 (17.5 mg), eluted with H_2_O/MeOH 40%, and identified as quercetin by ^1^H-NMR in comparison with the literature [44]. EA1, EA2 and EA3 NMR data are provided in the online Supplementary Material.

### 4.3. LC-MS Analyses of EA Fraction

The LC system, as previously described [45], was coupled to a mass spectrometer ESI-IT (Bruker Daltonics, Billerica, MA, USA), fitted with an electrospray ionization source operating in the positive mode, and an ion trap analyzer. The chromatographic conditions used were as follows: Luna C18 column (250 × 4.6 mm, 5 μm, Phenomenex, Torrance, CA, USA), sample injection volume of 10 μL at 1 mg/mL, flow rate 1.0 mL/min at 25 °C, and H_2_O containing 0.1% (*v*/*v*) formic acid (solvent A) and acetonitrile (solvent B) as the mobile phase. The elution gradient was 5% B in 0–3 min; 5%–10% B in 3–10 min; 10% B in 10–12 min; 10%–20% B in 12–15 min; 20% B in 15–17 min; 5%–10% B in 3–10 min; 10% B in 10–12 min; 10%–20% B in 12–15 min; 20% B in 15–17 min 35%–45% B in 32–40 min; 45% B in 40–42 min; 45%–60% B in 42–50 min; 60% B in 50–52 min; 60%–80% B in 52–60 min; 80% B in 60–62 min; 80%–100% B in 62–70 min; 100% B in 70–72 min; 100%–5% B in 72–75 min; 5% B in 75–76 min. The mass spectrometer parameters used were: capillary voltage, 3.5 kV; desolvation temperature, 330 °C; gas flow, 10 L/min; pressure, 70 psi, collision energy of 0.7 eV. Nitrogen was used as both the drying and nebulizing gas.

### 4.4. Preparation of Rat Aortic Rings for iSometric Tension Recording

All experimental protocols were approved by the Animal Care and Use Committee at Universidade Federal do Rio de Janeiro on 14 March 2012, under the license MACAÉ01. Thoracic aorta was dissected from male Wistar rats (200–250 g) and adipose and connective tissues were carefully removed. Aorta was cut into 3–4 mm rings, which were suspended in organ baths filled with Krebs-Henseleit solution (mM: 118.0 NaCl; 4.7 KCl; 1.2 KH_2_PO_4_; 1.2 MgSO_4_; 2.5 CaCl_2_; 25 NaHCO_3_ and 11.0 glucose; pH 7.4; 37 °C) continuously oxygenated with carborgen gas (95% O_2_, 5% CO_2_). Each aorta ring was mounted between two hooks in which one was attached to a force transducer (MLT0201; AD Instruments, Sydney, Australia), which signal was digitalized (Power Lab 4/30; AD Instruments) and stored on a computer for analysis using the software LabChart Pro (AD Instruments). After an equilibrium period of 1.5 h under 1 g resting tension, aortic rings were contracted with phenylephrine (Phe; 10 µM) and the presence of functional endothelium was confirmed by a relaxation response to acetylcholine (10 µM) greater than 80%. In some rings, the endothelium was mechanically removed, which was confirmed by the lack of relaxation in response to acetylcholine [46]. Concentration-response curves to MET, fractions and constituents were obtained in Phe-contracted rings.

In order to determine the involvement of NO pathway in vasodilatory activity of MET, aorta with endothelium were pretreated for 15 min with L-NAME (100 µM), an inhibitor of NO synthase; ODQ (100 µM), an inhibitor of soluble guanylyl cyclase (sGC); or wortmannin (300 nM), and inhibitor of phosphatidylinositol 3 kinase (PI3K). To verify the involvement of PGI_2_ pathway, aorta with endothelium was pretreated for 15 min with indomethacin (100 µM).

### 4.5. 1,1-Diphenyl-2-picrylhydrazyl (DPPH) Assay

The DPPH scavenging activity of MET and fractions was measured according to Nascimento et al. [47], with modifications. Methanolic solutions of plant extract and fractions at different concentrations (1–200 µg/mL) were mixed with a methanolic 300 µM DPPH solution in 96-wells microtiter plates and kept for 30 min at room temperature in the dark. Absorbances were measured at 517 nm (UVM 340 spectrophotometer, Biochrom ASYS, Cambridge, UK) using methanol as blank. *Ginkgo biloba* standardized extract of leaves (EGb 761^®^) was used as positive control. Absorbance values were converted into the percentage of antioxidant activity (AA%) by using the following formula: AA%= 100−{[(ABSsample – ABSblank) × 100] / ABScontrol}. To determine the concentration necessary to induce 50% of maximal response (EC_50_), results obtained from three separate experiments in triplicate were fitted by non-linear regression.

### 4.6. Statistical Analysis

Data are expressed as means ± S.E.M. Relaxation response is expressed as percentage of maximal tension observed in the presence of phenylephrine. Analyzes were performed using Prism 5.0 software (GraphPad Software, La Jolla, CA, USA). All data were analyzed using Kolmogorov-Smirnov normality test and all data have shown a Gaussian distribution. One-way analysis of variance followed by Newman-Keuls post-hoc test was used for comparison between concentration response curves. Differences between groups were considered statistically significant when *p* < 0.05.

## 5. Conclusions 

Our findings suggest that *T. guianensis* leaves could be a source of phenolic compounds with pharmacological potential, since MET could reduce vascular tone through NO-dependent vasodilation and reduce oxidative stress by its antioxidant activity. Tannins and flavonoids were identified in MET, and quercetin and myricetin glycoside seems to contribute for MET activity as they have been reported as vasodilatory flavonoids.

## Figures and Tables

**Table 1:** MS analyses of the *T. guianensis* EA fraction with emphasis on the major (254 nm chromatogram) and isolated compounds.

Retention Time (*t_R_*, min)	Identity	Pseudomolecular Ion *m*/*z*	Fragment *m/z* (Loss)	λ_MAX_ (nm)
18.9	1,4,6-tri-*O*-galloyl-β-d-glucose ^a^	-	467.12 (170)	276
21.0	quercetin 3-*O*-(6″-*O*-galloyl)-β-d-galactopyranoside ^a^	617.17 [M + H]^+^	-	263, 352
22.0	myricetin deoxyhexoside	465.13 [M + H]^+^	319.06 (146)	257, 350
23.9	quercetin pentoside	435.12 [M + H]^+^	303.05 (132)	256, 352
24.5	quercetin dideoxyhesoside	595.20 [M + H]^+^	449.13 (146) 303.06 (146)	255, 348
31.7	Quercetin ^a^	303.05 [M + H]^+^	-	255, 369

^a^ Isolated compound.

**Table 2:** MS analyses of *T. guianensis* EA active sub-fraction 4 with emphasis on major (254 nm chromatogram) and isolated compounds.

Retention Time (*t_R_*, min)	Identity	Pseudomolecular Ion *m*/*z*	Fragment *m/z* (Loss)	λ_MAX_ (nm)
18.9	1,4,6-tri-*O*-galloyl-β-d-glucose ^a^	-	467.08 (170)	277
19.1	galloyl-HHDP-hexoside	633.10 [M + H]^+^	-	266
21.0	quercetin 3-*O*-(6″-*O*-galloyl)-β-d-galactopyranoside ^a^	617.17 [M + H]^+^	-	263, 352
22.0	myricetin deoxyhexoside	465.13 [M + H]^+^	319.06 (146)	261, 350

^a^ Isolated compound.

**Table 3:** Antioxidant effect of MET and its fractions assessed by the DPPH assay.

Samples	EC_50_ (µg/mL)
MET	3.12 ± 0.20 ^a^
HN fraction	40.30 ± 0.39 ^c^
DCM fraction	19.83 ± 0.90 ^d^
EA fraction	5.33 ± 0.16 ^b^
BT fraction	6.05 ± 0.19 ^b^
Aq fraction	14.33 ± 0.15 ^e^
Egb 761^®^	22.91 ± 0.66 ^f^

EC_50_, Concentration required to induce 50% maximal response; MET, methanol extract of *T. guianensis* leaves; HN fraction, hexane fraction; DCM fraction, dichloromethane fraction; EA fraction, ethyl acetate fraction; BT fraction, butanol fraction; Aq fraction, aqueous fraction; Egb 761^®^, *Ginkgo biloba* extract). The results are means ± S.E.M. Statistical analyses were calculated and values with different superscript letters (^a–f^) are significantly different (*p* < 0.0001); determined by a Tukey test.

## Supplementary Materials

Supplementary materials are available online. ^1^H- and ^13^C-NMR spectroscopic data of the isolated compounds.
