# Supplementary material for: Phytochemical Study of Tapirira guianensis Leaves Guided by Vasodilatory and Antioxidant Activities
Source: Molecules. 2017 Feb 18;22(2):304. doi: 10.3390/molecules22020304 (PMC6155791; doi:10.3390/molecules22020304)
Supplement: Supplementary file 1 [file molecules-22-00304-s001.pdf]

Supplementary Materials: Phytochemical Study of *Tapirira guianensis* Leaves Guided by Vasodilatory and Antioxidant Activities

Amélia M. G. Rodrigues, Denise O. Guimarães, Tatiana U. P. Konno, Luzineide W. Tinoco, Thiago Barth, Fernando A. Aguiar, Norberto P. Lopes, Ivana C. R. Leal, Juliana M. Raimundo and Michelle F Muzitano

Phytochemical study of *Tapirira guianensis* leaves guided by vasodilatory and antioxidant activities

^{1}H- and ^{13}C-NMR spectra were recorded on a Variant spectrometer, MR-400 (^{1}H: 400 MHz; ^{13}C: 100 MHz) or VNMRSYS-500 (^{1}H: 500 MHz; ^{13}C: 125 MHz) using dimethyl sulfoxide-d_6 as solvent.

EA1 *(1,4,6-tri-O-Galloyl-β-D-glucose)*: ^{1}H-NMR (400 MHz, DMSO$_{d6}$): δ ppm 5.72 (d, *J* = 8.39 Hz, **H-1**); 3.43 (t, *J* = 8.39 Hz, **H-2**); 3.69 (t, *J* = 8.39 Hz, **H-3**); 5.02 (t, *J* = 8.39 Hz, **H-4**); 4.03 (d, *J* = 8.39 Hz, **H-5**); 4.21 (d, *J* = 12.28 Hz, **H-6**); 4.09 (dd, *J* = 12.28; 4.38 Hz, **H-6**); 6.96, 6.93, 7.03 (s, **H2′**, **6′**).

^{13}C-NMR (**100 MHz**, DMSO$_{d6}$): δ ppm 94.5 (**C-1**); 73.1 (**C-2**); 74.1 (**C-3**); 72.5 (**C-4**); 70.4 (**C-5**); 62.4 (**C-6**); 119.4, 119.4, 118.6 (**C-1′**); 109.3, 109.1, 109.4 (**C2′**, **6′**); 145.8, 145.9, 146.0 (**C3′**, **5′**); 139.0, 139.1, 139.6 (**C4′**), 165.3, 165.9, 164.9 (**C=O**).

EA2 *(Quercetin 3-O-(6-O-galloyl)-β-D-galactopyranoside)*: ^{1}H-NMR (500 MHz, DMSO$_{d6}$): δ ppm 6.17 (d, *J* = 1.96 Hz, **H-6**); 6.38 (d, *J* = 2.20 Hz, **H-8**); 7.51 (d, *J* = 2.20 Hz, **H-2′**); 6.81 (d, *J* = 8.31 Hz, **H-5′**); 7.64 (dd, *J* = 8.56, 2.20 Hz, **H-6′**); 5.33 (d, *J* = 7.83 Hz, **H-1′′**); 3.60 (dd, *J* = 9.5; 7.7 Hz, **H-2′′**); 3.45 (dd, *J* = 9.5; 3.2 Hz, **H-3′′**); 3.72 (m, **H-4′′**); 3.73 (m, **H-5′′**); 4.12 (dd, *J* = 10.64, 7.46 Hz, **H-6′′, a**); 4.04 (dd, *J* = 10.52, 5.87 Hz, **H-6′′**, **b**); 6.86 (s, **H-2′′′**, **6′′′**).
^{13}C-NMR (125 MHz, DMSO$_{d6}$): δ ppm 156.3 (**C-2**); 138.5 (**C-3**); 177.3 (**C-4**); 161.1 (**C-5**); 98.7 (**C-6**); 165.4 (**C-7**); 93.6 (**C-8**); 156.2 (**C-9**); 103.7 (**C-10**); 120.9 (**C-1′**); 115.9 (**C-2′**); 145.4 (**C-3′**); 148.6 (**C-4′**); 115.1 (**C-5′**); 121.9 (**C-6′**); 102.0 (**C-1′′**); 71.0 (**C-2′′**); 72.4 (**C-3′′**); 62.7 (**C-4′′**); 72.8 (**C-5′′**); 62.0 (**C-6′′**); 119.0 (**C-1′′′**); 108.5 (**C-2′′′, 6′′′**); 144.8 (**C-3′′′, 5′′′**); 138.5 (**C-4′′′**); 164.8 (**C=O**).

EA3 *(Quercetin)*: ^{1}H-NMR (400 MHz, DMSO$_{d6}$): δ ppm 6.15 (d, *J* = 1.96 Hz, 1 H; **H-6**); 6.37 (d, *J* = 1.96 Hz, 1 H; **H-8**); 6.85 (d, *J* = 8.51 Hz, 1 H; **H-5′**); 7.50 (dd, *J* = 8.51, 2.05 Hz, 1 H; **H-6′**); 7.64 (d, *J* = 1.96 Hz, 1 H; **H-2′**).
